# PPAR****γ**** Networks in Cell Signaling: Update and Impact of Cyclic Phosphatidic Acid

**DOI:** 10.1155/2013/246597

**Published:** 2013-02-07

**Authors:** Tamotsu Tsukahara

**Affiliations:** Department of Integrative Physiology and Bio-System Control, Shinshu University School of Medicine, 3-1-1 Asahi, Matsumoto, Nagano 390-8621, Japan

## Abstract

Lysophospholipid (LPL) has long been recognized as a membrane phospholipid metabolite. Recently, however, the LPL has emerged as a candidate for diagnostic and pharmacological interest. LPLs include lysophosphatidic acid (LPA), alkyl glycerol phosphate (AGP), cyclic phosphatidic acid (cPA), and sphingosine-1-phosphate (S1P). These biologically active lipid mediators serve to promote a variety of responses that include cell proliferation, migration, and survival. These LPL-related responses are mediated by cell surface G-protein-coupled receptors and also intracellular receptor peroxisome proliferator-activated receptor gamma (PPAR**γ**). In this paper, we focus mainly on the most recent findings regarding the biological function of nuclear receptor-mediated lysophospholipid signaling in mammalian systems, specifically as they relate to health and diseases. Also, we will briefly review the biology of PPAR**γ** and then provide an update of lysophospholipids PPAR**γ** ligands that are under investigation as a therapeutic compound and which are targets of PPAR**γ** relevant to diseases.

## 1. Introduction

**Figure 1:**
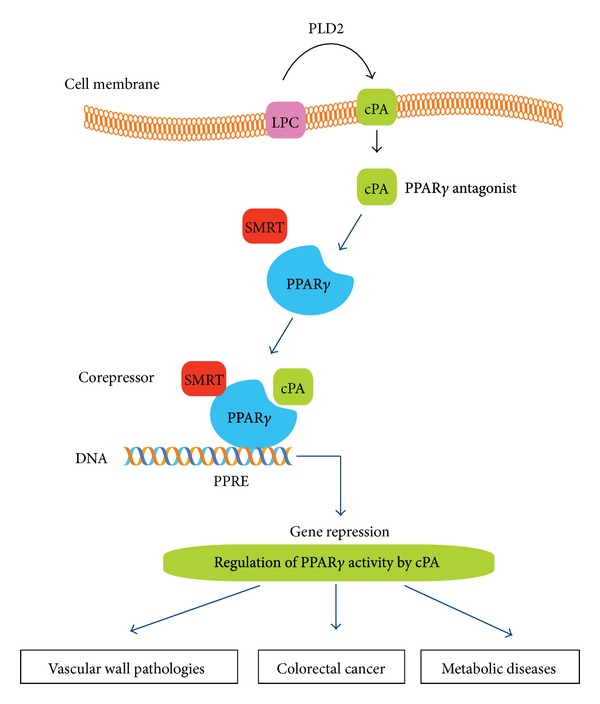
Regulation of PPAR*γ* activity by cPA. cPA is generated intracellularly in a stimulus-coupled manner by the PLD2 enzyme. cPA stabilize interactions with corepressor, such as SMRT, that act to repress gene transcription. This endogenous cPA regulates PPAR*γ* function required for vascular wall pathologies, colorectal cancer cell growth, and metabolic diseases.

Peroxisome proliferator-activated receptor gamma (PPAR*γ*) is a member of the nuclear hormone receptor superfamily, many of which function as ligand-activated transcription factors [1]. Synthetic agonists of PPAR*γ* include the thiazolidinedione (TZD) class of drugs, which are widely used to improve insulin sensitivity in type II diabetes. Despite the beneficial effects of PPAR*γ* on glucose and lipid homeostasis, excess PPAR*γ* activity can be deleterious. These classical PPAR*γ* agonists elicit a variety of side effects, including weight gain, edema, increased fat mass, and tumor formation in rodents [2]. In contrast, there have been many reports in which the putative physiological agonists of PPAR*γ* have been identified [3–5]. LPA is a naturally occurring phospholipid with growth-like effects in almost every mammalian cell type. LPAs elicit their biological responses through eight plasma membrane receptors [6] and intracellularly through the PPAR*γ* [3, 4]. Although LPA derived from hydrolysis of plasma membrane phospholipids is established as a ligand for G-coupled cell surface LPA receptor, studies suggested that LPA might also enter cells to activate PPAR*γ*. PPAR*γ* plays a role in regulating lipid and glucose homeostasis, cell proliferation, apoptosis, and inflammation [7, 8]. These pathways have a direct impact on human diseases in obesity, diabetes, atherosclerosis, and cancer [9–11]. On the other hand, cyclic phosphatidic acid (cPA), similar in structure to LPA, can be generated by phospholipase D2 (PLD2) and negatively regulate PPAR*γ* functions (Figure 1). cPA shows several unique actions compared to those of LPA. cPA inhibits cell proliferation, whereas LPA stimulates it [12–16]. It has been reported that cPA attenuates cancer cell invasion; moreover, metabolically stabilized derivative of cPA suppressed cancer cell metastasis [17, 18]. cPA is a second messenger and a physiological inhibitor of PPAR*γ*, revealing that PPAR*γ* is regulated by agonists as well as by antagonists.

## 2. Receptors and Signaling

### 2.1. Intracellular Receptor of PPAR*γ*

PPARs are members of the nuclear hormone receptor superfamily, many of which function as lipid-activated transcription factors [1]. There are three PPAR isoforms that include PPAR*α*, *β*/*δ*, and *γ* that differ in ligand specificity, tissue distribution, and developmental expression [19]. PPAR*γ*, the most extensively studied among the three PPAR subtypes, plays an important role in regulating lipid metabolism, glucose homeostasis, cell differentiation, and motility [10, 20]. There are 2 PPAR*γ* isoforms, PPAR*γ*_1_ and PPAR*γ*_2_. PPAR*γ*2 has 30 additional amino acids at the N-terminus in human caused by differential promoter usage and alternative splicing [21]. Genetic deletion of PPAR*γ*_1_ causes embryonic mortality [9]. In contrast, deletion of PPAR*γ*_2_ causes minimal alterations in lipid metabolism [22]. PPAR*γ*_1_ is expressed in almost all tissues, whereas PPAR*γ*_2_ is highly expressed in only the adipose tissue [21]. PPAR*γ* is comprised of four functional parts: the N-terminal A/B region bears a ligand-independent transcription-activating motif AF-1; C region binds response elements; D region binds to various transcription cofactors; and E/F region has an interface for dimerizing with retinoid X receptor *α* (RXR*α*), an AF-2 ligand-dependent transcription-activating motif, and a ligand binding domain (LBD) [23]. PPAR*γ* heterodimerizes with the retinoid X receptor *α* (RXR*α*), and it is the ligand binding domain (LBD) of PPAR*γ* that interacts with its agonists, including LPA [3]. The PPAR*γ*-RXR*α* heterodimer binds to the peroxisome proliferator response element (PPRE) in the promoter region of the target genes. In the absence of ligands, the corepressors, nuclear receptor corepressor (NCoR) and silencing mediator of retinoid (SMRT) and thyroid hormone, bind to the heterodimer to suppress the target gene activation [24]. Upon ligand binding, PPAR*γ* undergoes a conformational change that facilitates the dissociation of the corepressors and recruits coactivators. According to their mechanism of action, coactivators can be divided into two large families: the former includes steroid receptor coactivator (SRC-1) and CBP/p300, that act in part as molecular scaffolds and in the other part by acetylating divers substrates. The latter, including peroxisome proliferator-activated receptor 1*α* (PGC-1*α*), does not act by remodeling chromatin [25]. It has been reported that DNA methylation and histone modification serve as epigenetic markers for active or inactive chromatin [26]. A variety of putative physiological PPAR*γ* agonists have been identified [5, 27]. Since then, we and other authors have reported that selected forms of lysophospholipids, such as unsaturated LPA and alkyl glycerophosphate (AGP, 1-alkyl-2-hydroxy-sn-glycerol-3-phosphate), are physiological agonists of PPAR*γ* [3, 4]. The different molecular species of LPA contain either saturated or unsaturated fatty acids. Saturated LPA species including palmitoyl (16 : 0) and stearoyl (18 : 0) LPA are inactive. Among these ligands, AGP stands out with an equilibrium binding constant of 60 nM [4] that is similar to that of thiazolidinedione (TZD) class of synthetic agonists. Interestingly, some of the residues required for PPAR*γ* activation by AGP are different from those required by TZD drug. H323 and 449 within the LBD of PPAR*γ* are required for the binding and activation by rosiglitazone but are not required by AGP. R288 is an important residue for the binding of the AGP but not the rosiglitazone. Y273 is required for activation by both agonists [4]. AGP is unique in that its potency far exceeds that of LPA in activating PPAR*γ* [4]. The reason why AGP and unsaturated acyl-LPA species are the best activators of PPAR*γ* may reflect the differential delivery of these LPA analogs to PPAR*γ* versus saturated LPA species, which are inactive. Together, these data help to explain why PPAR*γ* binds the unsaturated LPA and AGP but not saturated LPA. On the other hand, we showed that cPA negatively regulates PPAR*γ* functions by stabilizing the SMRT-PPAR*γ* complex [15] and blocks TZD-stimulated adipogenesis and lipid accumulation. This ligand-dependent corepressor exchange results in transcriptional repression of genes involved in the control of insulin action as well as a diverse range of other functions. 

## 3. Targets of PPAR*γ* Relevant to Diseases

### 3.1. LPA-Mediated Activation of PPAR*γ* and Vascular Wall Pathologies

It has been reported that unsaturated LPA-elicited neointima was not mediated by the LPA GPCRs LPA_1_ and LPA_2_, which are the major LPA receptor subtypes expressed in the vessel wall [28]. LPA has been identified as a bioactive lipid and is produced in serum after the activation of multiple biochemical pathways [6, 29, 30]. Some clinical studies have shown the correlation between plasma LPA and vascular diseases [31]. Neointima formation is a characteristic feature of common vascular pathologies, such as atherosclerosis [32]. Atherosclerosis is a complex disease to which many factors contribute. Neointima lesions are characterized by accumulation of cells within the arterial wall and are an early step in the pathogenesis of atherosclerosis [33]. It is caused by a buildup of plaque in the inner lining of artery and made up of deposits of fatty substances and cholesterol [34]. Topical application of unsaturated LPA species into the noninjured carotid artery of rodents induces arterial wall remodeling [35, 36], and this response requires PPAR*γ*. PPAR*γ* plays an important role in the cardiovascular system. PPAR*γ* is expressed in all cell types of vessel wall, as well as monocytes and macrophages [37]. Macrophages play essential roles in immunity and lipid homeostasis [37]. PPAR*γ* is induced during the differentiation of monocytes into macrophages and is highly expressed in activated macrophages including the foam cells in atherosclerotic lesions [36]. CD36 is a one of PPAR*γ* response genes. PPAR*γ* activation upregulates CD36 expression which results in increased lipid uptake in macrophages [3]. In macrophages, oxidized low-density lipoprotein (oxLDL) uptake through CD36 results in the development of foam cells. Accumulation of foam cells in the arterial wall is a key event of the early atherosclerotic lesion [38]. The initial steps of foam cell formation have been extensively studied. A CD36-dependent signaling cascade is necessary for macrophage foam cell formation. Moore et al. reported that oxLDL uptake is decreased in PPAR*γ* deficient macrophages due to the loss of CD36 [39]. CD36 is a member of the class B scavenger receptor family of cell surface protein [38, 40]. These receptors are a group of receptors that recognize modified LDL by oxidation or acethylation [41]. It has been reported that LPA and AGP are an agonist of the PPAR*γ* and has been implicated in atherogenesis [36]. When AGP (18 : 1) was infused to an injured carotid artery, neointima thickening was augmented, although TZD drug, rosiglitazone- (Rosi-) attenuated neointima, induced by mechanical injury. However, noninjury model, Rosi, induces neointima when applied intraluminally into the carotid artery [15]. These results suggest that mechanisms underlying neointima formation in the chemically induced model are likely to be different from those in the injury-induced models. Coronary artery disease, the most common type of heart disease and leading cause of death among cardiovascular diseases, is almost always the result of atherosclerosis. Hence, the present results raise the possibility of utilizing this phospholipid scaffold as a lead for the development of new treatment acting on PPAR*γ*.

### 3.2. PPAR*γ* Ligand and Colorectal Cancer

Colon cancer is a malignancy that develops in colon and rectal tissues. Colon cancer cells can also spread to other parts of body. The prognosis for metastatic colon cancer is associated with high mortality [42, 43]. It has been reported that prognosis for metastatic colon cancer remains poor; therefore, new therapeutic options are needed to reduce cancer mortality. It has been reported that PPAR*γ* may provide a molecular link between a high-fat diet and increased risk of colon polyp formation during PPAR*γ* activation [44]. Two studies have shown that administration of a synthetic PPAR*γ* ligand to APC^Min/+^ mice resulted in these mice developing more frequent colon cancers than those animals which did not receive PPAR*γ* ligand [45]. APC^Min/+^ mice have a mutation of APC, which is a major regulator of *β*-catenin activation and represent a model of adenomatous polyposis coli (APC) [46]. Mutations of PPAR*γ* in colon cancer lead to the loss of ligand binding and suppression of cell growth. This may indicate that functional PPAR*γ* is required for the normal growth properties of colon cells [47]. The *PPAR*γ** gene is expressed in many tissues, including high levels of expression in normal colonic mucosa, colorectal adenocarcinomas, and colon cancer cell lines [11, 48]. Recently, several studies reported that PPAR*γ* agonists inhibit cancer cell proliferation, survival, and invasion [16, 49]. Although PPAR*γ* is expressed at significant levels in human colon cancer cells and tissues, the role of PPAR*γ* activation in colon cancer is still controversial. Furthermore, the role of PPAR*γ* activation in cancer remains unclear. Some reports indicate that PPAR*γ* is expressed at considerable levels in human colon cancer cells and tissues and that treatment with PPAR*γ* agonists and antagonists reduces the cell growth rate [16, 50, 51]. Because PPAR*γ* ligands have been shown to have a variety of PPAR*γ*-dependent and -independent effects [52]. Our recent reports suggest that endogenous LPA agonist, cPA, which is a *bona fide* second messenger and a physiological inhibitor of PPAR*γ* [15] has emerged as a potential therapeutic target in the treatment of colon cancer [16]. cPA is a structural analog of LPA, which is one of the simplest phospholipids in cells. cPA is a generated by phospholipase D-catalyzed-transphosphatidylation of lysophosphatidylcholine (LPC) [15]. LPA is a PPAR*γ* agonist that induces cell proliferation and invasion, but cPA exerts the opposite effects in cancer cells [16]. cPA suppresses PPAR*γ* activation both by preventing binding of exogenous agonist to PPAR*γ* and by inducing a specific conformational change that suppresses PPAR*γ* activation [15, 16, 53]. cPA binding to and inhibition of PPAR*γ* might be involved in cPA-induced inhibition of colon cancer cell growth [16]. This study demonstrates the potential applications of these methods for colon cancer treatment. 

### 3.3. cPA in the Treatment of Metabolic Diseases

Obesity and its associated conditions such as insulin resistance, type II diabetes, termed as the metabolic syndrome, is a worldwide health problem and occurs as a result of adipose tissue enlargement caused by store excess energy intake [54]. Obesity is a condition in which adipocytes accumulate a large amount of body fat and became enlarged [55]. Adipose differentiation is a complex process by which fibroblast-like undifferentiated cells are converted into cells that accumulate lipid droplets [56]. PPAR*γ* agonists are known to induce the differentiation of preadipocytes into mature adipocyte. TZD drugs are widely used in type II diabetes mellitus to improve insulin sensitivity by inducing the expression of genes involved in adipocyte differentiation, lipid and glucose uptake, and fatty acid storage [19]. Our recent observation suggests that PPAR*γ* activation in adipogenesis that can be blocked by treatment with cPA participates in adipocyte function through inhibition of PDE3B expression [57]. cPA reduced intracellular triglyceride levels and inhibited the phosphodiesterase 3B (PDE3B) expression in 3T3-L1 adipocytes [57]. Treatment of 3T3-L1 cells with cPA significantly increased the amount of free glycerol. This suggests that triglyceride was hydrolyzed in adipocytes to free fatty acid and glycerol through the lipolysis. Adipose tissue lipolysis is dependent on the intracellular concentration of cAMP [58, 59], which is determined at the levels of both synthesis and degradation. Hydrolysis of cAMP is accomplished by PDEs [60]. Investigation on PDE has been focused on hormone-sensitive PDE3B activity and its expression. PDE3B is expressed in insulin-sensitive cells and has been shown to be important in regulating antilipolysis [61]. These findings contribute to the participation of cPA on the lipolytic activity in adipocytes. cPA might be a therapeutic compound in the treatment of obesity and obesity-related diseases including type II diabetes and high blood pressure. 

## 4. Conclusion

Clearly, the genomic response to activation and inhibition of PPAR*γ* is complex and will be highly dependent on cellular context. PPAR*γ* agonists and antagonists participate in the regulation of lipid metabolism, they play an important role during atherosclerosis, diabetes, and they also have a critical role in the regulation of growth of cancer cells. It has been suggested that PPAR*γ* ligands with agonistic and antagonistic effects may have useful role in the treatment of PPAR*γ*-mediated diseases. We can expect many promising results in this area in the near future.
